# Prone positioning monitored by electrical impedance tomography in patients with severe acute respiratory distress syndrome on veno-venous ECMO

**DOI:** 10.1186/s13613-020-0633-5

**Published:** 2020-02-03

**Authors:** Guillaume Franchineau, Nicolas Bréchot, Guillaume Hekimian, Guillaume Lebreton, Simon Bourcier, Pierre Demondion, Loïc Le Guennec, Ania Nieszkowska, Charles-Edouard Luyt, Alain Combes, Matthieu Schmidt

**Affiliations:** 1 INSERM, UMRS_1166-iCAN, Institute of Cardiometabolism and Nutrition, Sorbonne Universités, UPMC Univ Paris 06, 75651 Paris Cedex 13, France; 2 Medical Intensive Care Unit, Assistance Publique-Hôpitaux de Paris, Pitié-Salpêtrière Hospital, 75651 Paris Cedex 13, France; 3 Cardiac Surgery Department, Assistance Publique-Hôpitaux de Paris, Pitié-Salpêtrière Hospital, 75651 Paris Cedex 13, France

**Keywords:** Acute respiratory distress syndrome, Prone position, Electric impedance tomography, Extracorporeal membrane oxygenation

## Abstract

**Background:**

Prone positioning (PP) during veno-venous ECMO is feasible, but its physiological effects have never been thoroughly evaluated. Our objectives were to describe, through electrical impedance tomography (EIT), the impact of PP on global and regional ventilation, and optimal PEEP level.

**Methods:**

A monocentric study conducted on ECMO-supported severe ARDS patients, ventilated in pressure-controlled mode, with 14-cmH_2_O driving pressure and EIT-based “optimal PEEP”. Before, during and after a 16-h PP session, EIT-based distribution and variation of tidal impedance, VT_dorsal_/VT_global_ ratio, end-expiratory lung impedance (EELI) and static compliance were collected. Subgroup analyses were performed in patients who increased their static compliance by ≥ 3 mL/cmH_2_O after 16 h of PP.

**Results:**

For all patients (*n* = 21), tidal volume and EELI were redistributed from ventral to dorsal regions during PP. EIT-based optimal PEEP was significantly lower in PP than in supine position. Median (IQR) optimal PEEP decreased from 14 (12–16) to 10 (8–14) cmH_2_O. Thirteen (62%) patients increased their static compliance by ≥ 3 mL/cmH_2_O after PP on ECMO. This subgroup had higher body mass index, more frequent viral pneumonia, shorter ECMO duration, and lower baseline VT_dorsal_/VT_global_ ratio than patients with compliance ≤ 3 mL/cmH_2_O (*P* < 0.01).

**Conclusion:**

Although baseline tidal volume distribution on EIT may predict static compliance improvement after PP on ECMO, our results support physiological benefits of PP in all ECMO patients, by modifying lung mechanics and potentially reducing VILI. Further studies, including a randomized–controlled trial, are now warranted to confirm potential PP benefits during ECMO.

## Introduction

Despite improvements in the management of severe acute respiratory distress syndrome (ARDS), in-hospital mortality remains high, exceeding 40% [1]. Some patients with severe ARDS and refractory hypoxemia, hypercapnia or uncontrolled high airways pressures may benefit from venovenous-extracorporeal membrane oxygenation (VV-ECMO) [2]. One of the main goals of VV-ECMO is to rest the lungs by applying a so-called “ultra-protective” ventilation strategy, combining significant reductions of the tidal volume (VT) and intrathoracic pressures [3, 4], to enhance prevention of ventilator-induced lung injury (VILI).

Prone positioning (PP) is an effective first-line intervention to treat ARDS [5], as it improves gas exchanges [6] and lowers mortality [7]. However, the response to PP remains unpredictable [8, 9] and may differ from one patient to another [7, 10]. Despite these differences, PP is associated with greater survival [10]. This effect on mortality regardless of impact on respiratory mechanics could be explained by a more uniform distribution of VT leading to a reduction of VILI [11], but this effect was not well studied.

Although the use of PP combined with ECMO is still controversial [12], few studies have suggested that it could improve oxygenation and static compliance [13, 14], thereby preventing subsequent VILI, when associated with “ultra-protective” ventilation. To date, PP physiological effects on regional ventilation and the optimal positive end-expiratory pressure (PEEP) level in this specific population with very low VT and altered static compliance are unknown. Moreover, due to the extreme severity of these patients, and the inherent risks of PP in this specific population, such as decannulation, teams could be reluctant to perform PP.

In that context, electrical impedance tomography (EIT) could be a promising tool to describe the respective impacts of PP on regional ventilation and possible change of the “optimal PEEP” level on PP. Indeed, EIT is bedside, real-time, non-invasive, functional and radiation-free imaging of the lungs, which provides a regional dynamic lung analysis [15, 16]. Its performance in the context of ECMO-supported severe ARDS was validated, showing that it could be a useful tool to titrate the optimal PEEP in this severely ill population [17].

We hypothesized that EIT could help to monitor the impact of PP on regional ventilation and optimal PEEP highlighting potential beneficial effects to prone ECMO-treated severe ARDS patients. The aims of our study on ECMO-supported severe ARDS patients were therefore to describe (1) the PP impact on regional ventilation; (2) the PP influence on the optimal PEEP level; and (3) to identify different EIT patterns depending on static compliance gain.

## Methods

### Study design and procedure

We conducted this study during a 4-month period in our 26-bed medical intensive care unit (ICU). Written informed consent was obtained from all patients’ surrogates before inclusion. The study was approved by the appropriate legal and ethical authorities (Comité de Protection des Personnes Sud-Est VI, Clermont-Ferrand, France, AU1431).

### Patients

Inclusion criteria were: ARDS patient on VV-ECMO, pressure-controlled mechanical ventilation mode and sedated with a Richmond Agitation–Sedation Scale ≤ − 2. Exclusion criteria mainly reflected clinical contraindications to using EIT, PP or high PEEP levels (details are provided in the Additional file 1).

### Protocol

**Fig. 1:**
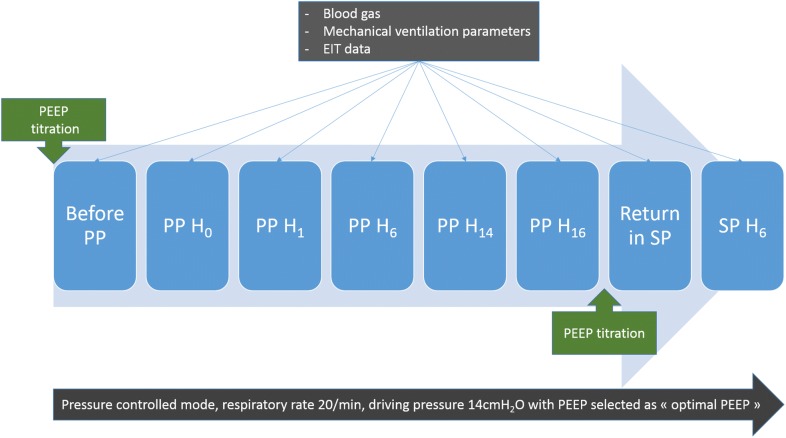
Study protocol. *EIT* electrical impedance tomography, *PEEP* positive end-expiratory pressure, *PP* prone positioning, *PP H*_*0*_ immediately after prone positioning, *SP* supine positioning

The different steps of the protocol are described in Fig. 1. To prevent spontaneous breathing, patients were fully sedated by continuous infusion of propofol, sufentanil, and atracurium. All patients were ventilated with a V500 ventilator (Dräger, Lübeck, Germany) in pressure-controlled mode with a constant driving pressure (plateau pressure minus total PEEP) of 14 cmH_2_O [1, 18] with the respiratory rate set at 20/min. Total PEEP was obtained after an end-expiratory pause. To ensure that measured peak pressure accurately reflected plateau pressure, we verified that inspiratory flow was zero at the end of inspiration.

#### PEEP titration

“Optimal PEEP” was identified, by PEEP titration, in supine positioning (SP), and after 16 h of PP. To reach a steady-state, we first performed a recruitment maneuver, applying 40 cmH_2_O for 40 s [19]. Then, a decremental PEEP trial was performed starting from 20 and decreasing to 6 cmH_2_O in 2-min steps of 2 cmH_2_O. Driving pressure was maintained constant at 14 cmH_2_O. Overdistention and collapse percentages at each PEEP level were calculated, as previously described [17, 20]. Thus, optimal PEEP, i.e., the lowest sum of collapse and overdistension percentages [17], was identified and applied throughout the protocol. A similar PP PEEP trial was repeated after 16 h of PP (i.e., just before returning on supine) to compare their optimal PEEP levels. In case of similar results for 2 different PEEP levels, the one with collapse to 15% or less while maintaining the lowest percentage of overdistention was selected [17].

#### PP

Briefly, patients were ventilated at previously estimated optimal PEEP with constant driving pressure (14 cmH_2_O) and a constant respiratory rate of 20/min. PP lasted 16 h. EIT data were recorded before PP, immediately after PP, after 1, 6, 14 and 16 h of PP, respectively, after returning to SP, and after 6 h in SP. A recruitment maneuver, consisting of sustained inflation at 40 cmH_2_O for 40 s [19], was performed at each step before recording EIT data.

#### Data acquisition

At each step, hemodynamic parameters, VT, corrected minute ventilation (minute ventilation × PaCO_2_/40) [21], arterial blood gases and EIT data were recorded. Global static compliance (henceforth referred to as compliance) was calculated by dividing expiratory VT by 14 cmH_2_O (i.e., constant driving pressure). Lastly, fluid balance throughout the protocol was noted.

#### EIT acquisition and analyses

EIT analysis is detailed in the Supplemental Digital Content. Briefly, lung images were divided into two symmetrical non-overlapping ventral and dorsal horizontal regions of interest (ROIs) [15]. The vertical height of these ROIs was the same, corresponding to 50% of the anteroposterior diameter. At each protocol step, we recorded impedance variation (Δz) (i.e., well-correlated with VT [15]), local compliance variation [20], which is calculated dividing local volume variation (based on impedance variation obtained with EIT) by the driving pressure, VT_dorsal_/VT_global_ (i.e., expressed as the percentage of Δz located in dorsal regions) [22–25], and end-expiratory lung impedance (EELI) (i.e., well-correlated with variations of end-expiratory global and regional lung volumes [26]). Notably, because the relationship between EELI and end-expiratory global and regional lung volumes is not linear [26], EELI cannot be accurately transposed into regional or global end-expiratory lung volumes values. Out-of-phase regions (with a decreased of impedance during inflation, leading to negative values of Δz), were excluded of analysis [27, 28] (details are provided in the Supplemental Digital Content).

#### Evaluation of PP response

Because it would have been difficult to discern the direct PP influences on gas exchanges under ECMO, we arbitrarily decided not to use PaO_2_ or PaCO_2_ as key markers to identify effects of PP on patients. We rather chose to analyze the PP impact on respiratory mechanics, using compliance to evaluate PP response. We performed a subgroup analysis differentiating patients with a compliance gain (PCG+) after 16 h of PP, defined by an increase ≥ 3 mL/cmH_2_O compared to baseline (i.e., SP) [9, 10, 29], and patients with no compliance gain (PCG−). We arbitrarily chose that definition by considering that a VT increase of > 40 mL after PP would be more likely to represent a “true” compliance gain rather than an inherent measurement variability, which could not be excluded with a smaller VT increase on ECMO. Consequently, a compliance increase of ≥ 3 mL/cmH_2_O with driving pressure at 14 cmH_2_O would result in a VT gain of ≥ 42 mL.

### Statistical analyses

Statistical analyses were computed with Prism 4.01 software (GraphPad Software, San Diego, CA). Because all parameters had non-normal distributions, data are expressed as median (interquartile range; IQR). Friedman ANOVA for repeated measures was used to compare data obtained at each step, followed, when appropriate, by pairwise comparisons using a Dunn post hoc test with Bonferroni correction. Qualitative data were compared with Fisher’s exact test. All comparisons were two-tailed, with *P* < 0.05 required to assert the presence of a difference.

## Results

### Population

Twenty-one patients (median (IQR) age 56 (46–61) years, 62% male) were included; their main characteristics are reported in Table 1. Briefly, the most frequent ARDS-risk factors were viral (57%) and bacterial pneumonia (19%). Patients were included after 8 (6–11) days on mechanical ventilation, and 2 (1–5) days on ECMO, with 76% having at least one PP session before cannulation.

**Table 1:** Characteristics and outcomes according to PP-responder status

Characteristic	All patients (*n* = 21)	PP responders (*n* = 13)	PP non-responders (*n* = 8)	*P*
Age, year	56 (46–61)	59 (45–62)	49 (36–55)	0.07
Male, *n* (%)	13 (62)	8 (62)	5 (63)	0.68
BMI (kg/m^2^)	29 (27–39)	30 (29–40)	27 (23–34)	0.046
SAPS II	68 (55–79)	70 (52–76)	66 (57–87)	0.59
SOFA	13 (11–16)	13 (11–16)	14 (12–16)	0.64
ARDS-risk factor				
Viral pneumonia	12 (57)	9 (69)	3 (38)	0.004
Bacterial pneumonia	4 (19)	4 (31)	0	
Aspiration pneumonia	2 (10)	0	2 (26)	
Other	3 (14)	0	3 (38)	
MV duration before inclusion (d)	8 (6–11)	7 (5–9)	12 (5–28)	0.13
Tidal volume (mL/kg)	4.2 (3.3–5.4)	5.1 (3.4–5.9)	3.6 (2.7–5.4)	0.12
Static compliance (mL/cmH_2_O)	22.6 (18.1–28.9)	23.8 (18.3–33.4)	20.1 (8.7–28.0)	0.26
“Optimal PEEP” before PP (cmH_2_O)	14 (12–16)	14 (12–17)	11 (8–15)	0.11
PP before inclusion (%)	16 (76)	9 (69)	7 (88)	0.61
Median number of PP sessions	2 (1, 2)	3 (2–4)	2 (1–6)	0.85
ECMO duration before inclusion (d)	2 (1–5)	3 (1–5)	1 (1–18)	0.65
ECMO flow (L/min)	5.1 (4.3–5.6)	5.1 (4.3–5.6)	5.1 (4.3–5.7)	0.89
ECMO sweep-gas flow (L/min)	5 (3–8)	5 (3–7)	5 (3–8)	0.59
Fluid balance, mL/24 h	480 (− 710 to 1037)	600 (− 930 to 1100)	1100 (− 180 to 2700)	0.80
MV duration (d)	43 (27–62)	34 (27–55)	59 (46–82)	0.06
ECMO duration (d)	16 (11–23)	13 (10–19)	28 (13–65)	0.046
ICU Length of stay (d)	58 (59–71)	42 (28–64)	69 (59–92)	0.02
In-ICU deaths (%)	8 (38)	5 (38)	3 (38)	1

Results are expressed as mean/median (IQR). *ARDS* acute respiratory distress syndrome, *BMI* body mass index, *d* days, *ECMO* extracorporeal membrane oxygenation, *ICU* intensive care unit, *MV* mechanical ventilation, *PEEP* positive end-expiratory pressure, *PP* prone positioning, *SAPS II* Simplified Acute Physiology Score II, *SOFA* Sequential Organ-Failure Assessment

### Static global compliance, local compliance variation and gas exchanges

**Fig. 2:**
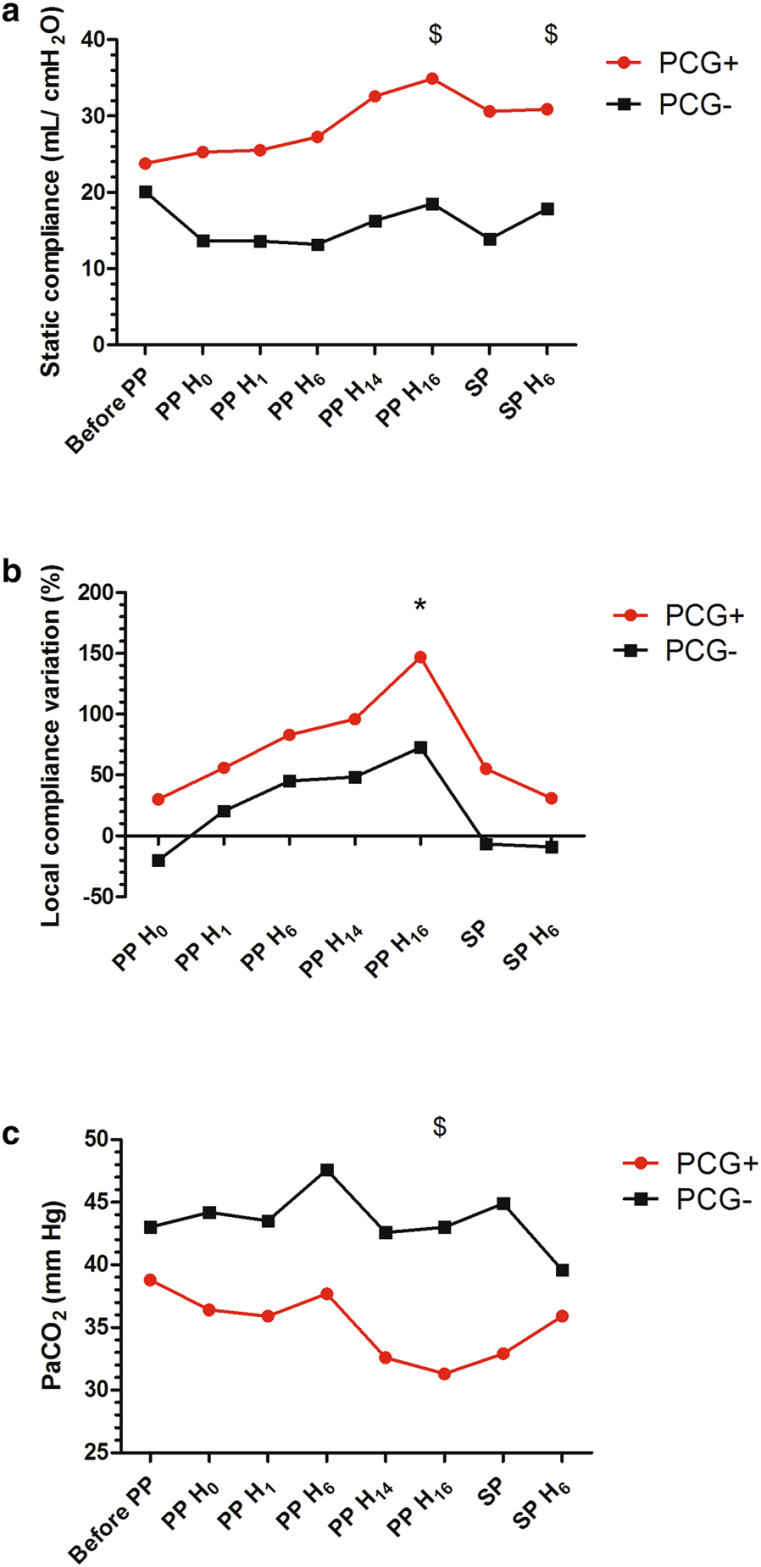
**a** Trend of median static compliance variation; **b** median local compliance variation compared to baseline (expressed in percentage); and **c** impact of prone positioning on median PaCO_2_ before, during, and after prone position for patients with an increase of the static compliance by ≥ 3 mL/cmH_2_O (PCG+) (red) and patients with static compliance < 3 mL/cmH_2_OsPP after 16-h of PP (PCG−) (black). PP: prone positioning; SP: supine positioning; *p < 0.005 vs. baseline for both PCG+ and PCG− patients; $*p* < 0.005 vs. baseline for PCG+ patients only

Static compliance during PP session increased from 23 (17–29) to 27 (20–37) mL/cmH_2_O (*P* < 0.01), corresponding to a VT and a corrected minute ventilation increase from 4.2 (3.3–5.4) to 5.6 (3.8–6.4) mL/kg and 5.5 (4.3–6.8) to 6.2 (5.1–6.9) L/min (*P* = 0.04), respectively. Consequently, local compliance variations, which is calculated based on local impedance variation compared to baseline, increased by 120 (60–180) % at the end of PP (Fig. 2). Notably, 62% of patients increased their static compliance by more than 3 mL/cmH_2_O (PCG+). Their global static compliance increased from 25 (19–31) to 35 (27–39) mL/cmH_2_O, resulting in an increase of the VT and the corrected minute ventilation from 5.1 (3.4–5.9) to 6.3 (5.6–6.7) mL/kg, and from 6.2 (4.8–6.8) to 6.6 (5.3–7.4) L/min, respectively. These benefits remained significant 6 h after return in SP (*P* ˂ 0.0001) (Fig. 2). Interestingly, they exhibited a significant decrease in PaCO_2_ from 39 (34–41) mmHg to 31 (29–37) mmHg after a PP session (p = 0.03) without any variation of sweep gas flow (Fig. 2). They significantly more frequently had viral pneumonia and higher body mass index than patients without static compliance gain. In addition, PCG+ required shorter ECMO duration and ICU length of stay than PCG−.

### Tidal impedance variation and EELI

**Fig. 3:**
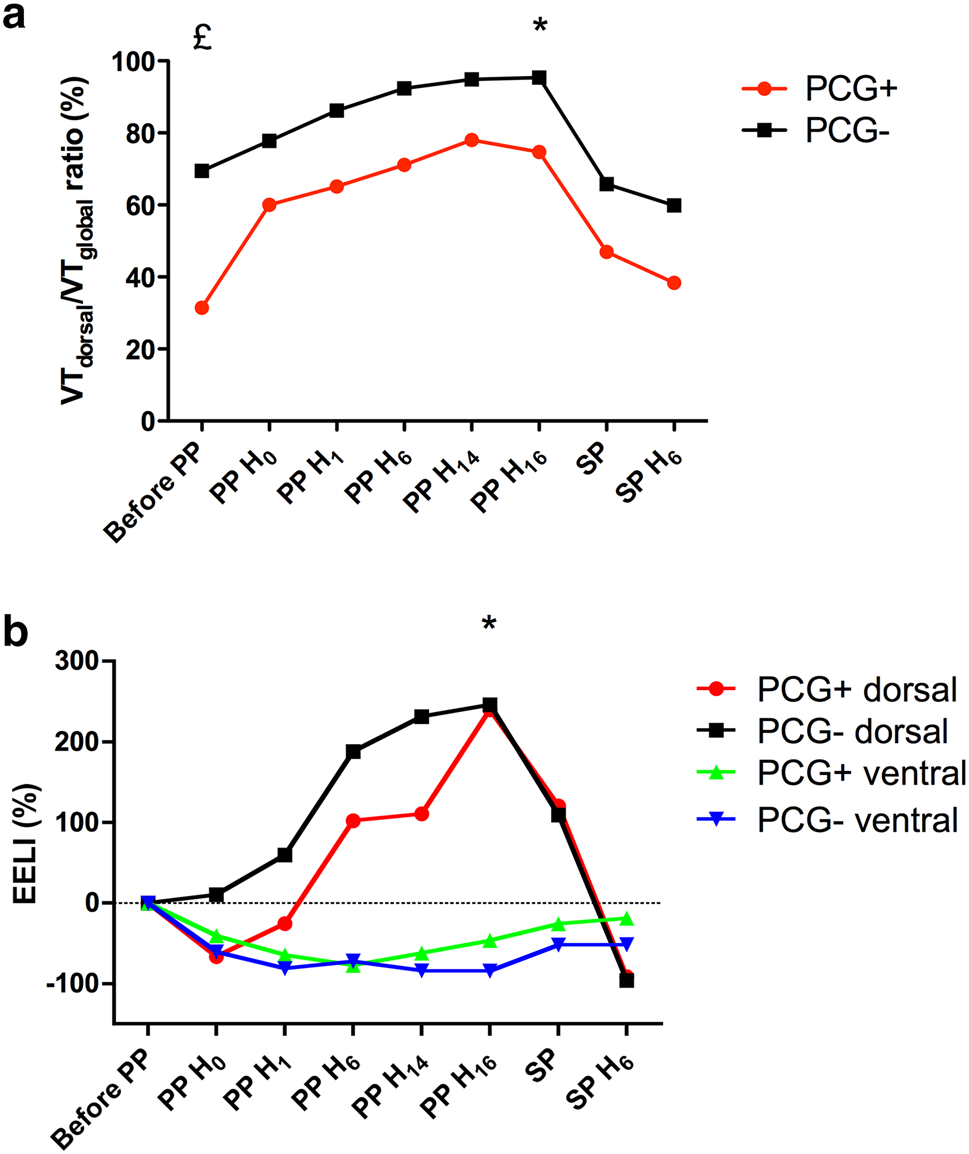
**a** Variation of the median VT_dorsal_/VT_global_;** b** variation of EELI compared to baseline in dorsal and ventral regions, for patients with an increase of the static compliance by ≥ 3 mL/cmH_2_O (PCG+)) and patients with static compliance < 3 mL/cmH_2_O, respectively (PCG−). *p < 0.05 vs. baseline; £p < 0.05 between PCG+ and PCG−

**Fig. 4:**
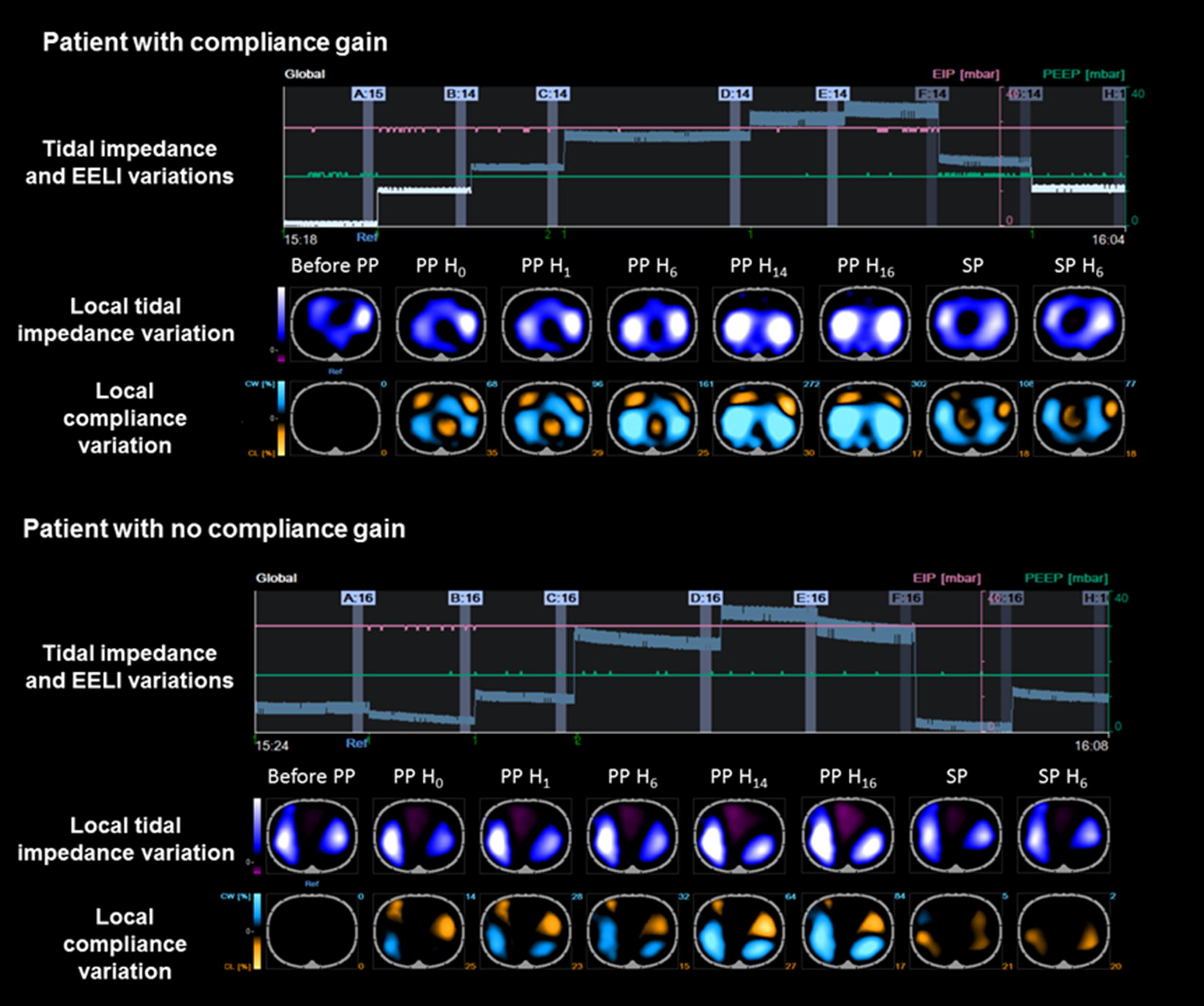
Summary of EIT findings obtained during a prone-positioning (PP) session in two representative patients, a patient with an increase of the static compliance by ≥ 3 mL/cmH_2_O after 16-h of PP and a patient with static compliance ˂3 mL/cmH_2_O, respectively. *EELI* end-expiratory lung impedance, *H* hour. For graphic illustration of tidal impedance variation (∆impedance), the lighter the zone, the greater the ∆impedance, meaning that ∆impedance is higher in white zones than blue zones. For local compliance variation distribution, blue regions reflect a compliance gain compared to baseline (i.e., before PP), whereas yellow regions represent a compliance loss

After 16 h of PP, tidal impedance shifted significantly to the dorsal ROI for all patients, regardless of the impact of PP on global compliance (*P* < 0.05), with VT_dorsal_/VT_global_ ratio significantly higher after the 16-h PP session versus baseline (Fig. 3). In addition, EELI located in the dorsal ROI increased by 240 (− 52 to 666)%, while EELI located in ventral ROI decreased by 81 (− 156 to 107)% after 16 h of PP (Fig. 3). Figure 4 shows representative EIT findings before, during and after a PP session for PCG+ and PCG−. The ventilation distribution before PP differed significantly between these two subgroups, with respective median VT_dorsal_/VT_global_ ratio values of 31 (29–48) and 69 (36–76)% (*P* < 0.05) (Fig. 3). That finding illustrated the predominant pre-PP VT distribution in the ventral regions of PCG+ , while it was more likely distributed in the dorsal regions of PCG−.

### EIT-identified optimal PEEP

**Fig. 5:**
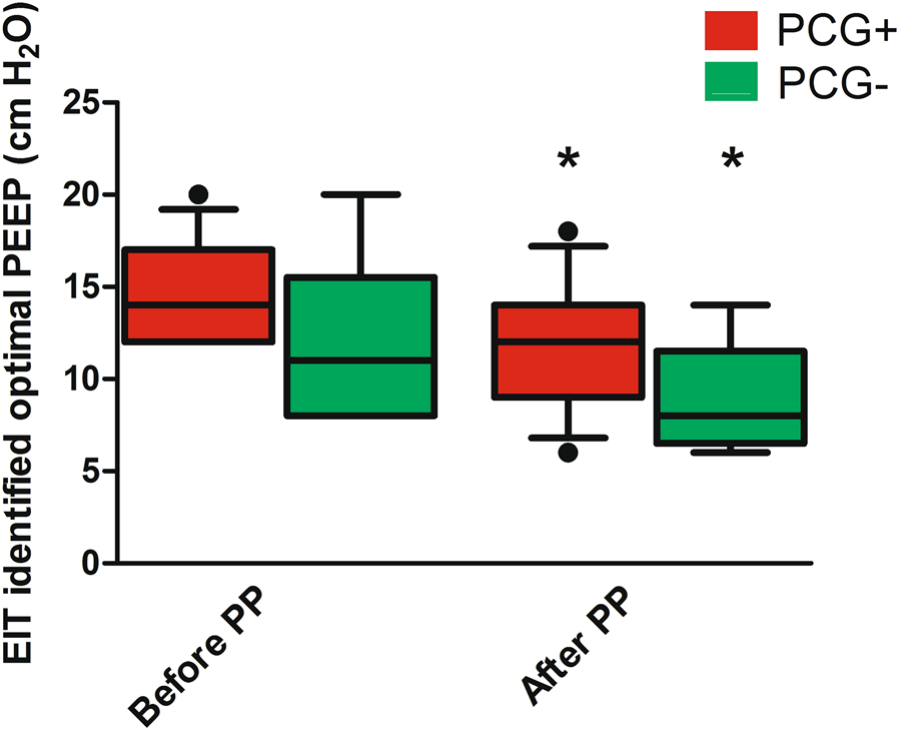
Electrical impedance tomography (EIT)-estimated optimal positive end-expiratory pressure (PEEP) before and at the end of a prone positioning (PP) session, for patients with an increase of the static compliance by ≥ 3 mL/cmH_2_O (PCG−) (red) and patients with static compliance < 3 mL/cmH_2_O, respectively (PCG−) (green). Whiskers plots report the median = central horizontal line; interquartile range = bottom and top box limits and 90% percentile = T bars, • = outliers. *P < 0.05 vs. baseline

Notably, EIT-identified optimal PEEP before PP did not differ significantly between PCG+ and PCG− (*P* = 0.09). It was significantly reduced after PP in all patients, shifting from 14.8 (12–17) to 11.7 (9–14) cmH_2_O for PCG+ , while PCG−, optimal PEEP declined from 12 (8–15.5) to 9 (6.5–11.5) cmH_2_O (Fig. 5).

## Discussion

To our knowledge, PP physiological effects in a population of patients with severe ARDS on ECMO have not yet been thoroughly studied. Based on EIT-monitoring, the main findings were: (1) although PCG+ and PCG− had different tidal volume distribution before PP, all patients with severe ARDS on ECMO exhibited a shift of the VT distribution and EELI from the ventral-to-dorsal ROIs resulting in an increased of the local compliance and the VT_dorsal_/VT_global_ ratio and; (2) EIT-estimated optimal PEEP decreased with PP, highlighting the potential reduction of atelectasis with PP.

By defining PP “responders” on PaO_2_ improvement, other ventilatory monitoring tools, such as thoracic tomodensitometry) [8] or lung ultrasonography [9], have failed to predict PP response at baseline. Because we anticipated that gas exchanges may be more likely influenced by ECMO settings than PP effects, we chose to use modification of static compliance to identify two profiles of PP response. Interestingly, 62% patients were classified as being PCG+ . Their PP sessions were associated with a significant decrease of PaCO_2_, which is, a well-known marker of PP response [10, 30]. In addition, this subgroup had higher body mass indexes and more frequent viral pneumonia, highlighting the potential benefits to pursue PP in these patient subgroups.

These data suggest that EIT could offer a promising bedside, dynamic, non-invasive, functional analysis of lung mechanics that could predict and monitor potential PP “response” on ECMO. Our results mainly underscored immediate PP effects, which continued to evolve during the 16-h procedure, illustrating the need for long PP sessions to obtain the best benefits [6, 31, 32]. Consistent with previous publications [13, 14], we found that on-ECMO PP was simple, feasible and safe with no PP-related complications of the ECMO circuit.

Baseline lung mechanics and predominant lesions differed between PCG+ and PCG− patients, and could explain these different responses between subgroups. Indeed, baseline tidal impedance were mostly distributed in ventral ROIs only in PCG+ . This finding suggests a collapse of the dorsal lung ROIs and functional aerated ventral ROIs in PCG+ , whereas ventral ROIs were over distended in PCG− at baseline. Consistently, previous observations reported that PaCO_2_-based beneficial PP effects mainly depend on the lung recruitment/derecruitment ratio [11, 33]. In addition, EIT-determined local compliance increased at a higher percentage than global compliance, suggesting a potential negative PP impact on another lung ROI, not captured by EIT at this thoracic level. Pertinently, Bikker et al. described different EIT-pattern responses to a PEEP trial when they were evaluated at two different thoracic levels. Hence, it cannot be excluded that different response patterns at different thoracic levels might also occur during PP.

Based on these results, should we decide to perform PP based on predicted PP-response for ECMO patients? The relevance of this question appears low. Indeed, the increase of EELI in the dorsal regions was observed in all patients, as previously reported with other tools [34, 35] and in other populations. Moreover, it is worth noting that PP significantly impacted on regional VT distribution and optimal PEEP levels in all patients, regardless of their static compliance modifications. Our study suggests that global static compliance or gas exchanges are not good surrogate of the impact of PP on regional ventilation. Indeed, improvement of local compliance, VT and EELI redistribution, were observed even for patients with lower global static compliance at the end of PP. Our preliminary results suggest striving to prone all ECMO-supported ARDS patients regardless their predicted response in terms of static compliance improvement. These results are consistent with previous studies suggesting that PP benefits are independent of the oxygenation/decarboxylation responses [10] and may be more related to less VILIs.

One of our main results is the impact of PP on optimal PEEP evaluated with EIT. EIT-based optimal PEEPs decreased significantly after 16 h of PP, highlighting the remarkable PP impact on respiratory mechanics. To date, this aspect of PP management has only been studied in patients with healthy lungs and yielded controversial results. Spaeth et al. [36] reported that PP was required to increase PEEP to avoid lung collapse in patients with healthy lungs after lumbar spine surgery, whereas Petersson et al. [37] suggested that application of PEEP during PP was associated with increased ventilation/perfusion mismatch in healthy subjects.

Our study has several limitations. First, EIT provides only a cross-sectional lung-region evaluation, which may differ from whole lungs [22]. This distinction might explain the differences between local and global compliance variations reported herein. Second, we chose to apply a 16-h PP session, as described by Guerin et al. [7]. However, our findings cannot rule out that the PP impact could evolve beyond 16 h, enhancing the PP impact on local and global ventilation. That hypothesis was also supported by Kimmoun et al.’s observations, after subjecting 17 severe ARDS patients on VV-ECMO to one or more 24-h PP sessions [14]. In this study, patients exhibited major compliance improvement that persisted 24 h after returning to SP. Third, we defined optimal PEEP as the best compromise between lowest overdistension and collapse, as previously described [20]. We cannot exclude that different optimal PEEP identification methods, e.g., inhomogeneity index [38] or dependent silent spaces [39], would have yielded different results. However, we can acknowledge that the selected optimal PEEP’s influence on our results was probably limited because ventilator settings were unchanged throughout the entire protocol. Lastly, our study enrolled relatively few patients and our promising results need to be confirmed in larger studies.

## Conclusion

EIT monitoring of PP during VV-ECMO shows that PP impacts global and regional ventilation, illustrated by a progressive redistribution of VT and EELI from ventral to dorsal regions. Although baseline VT distribution on EIT may predict static compliance improvement after PP on ECMO, our results support physiological benefits of PP in all ECMO patients, by modifying lung mechanics and potentially reducing VILI. In addition, clinicians should be also encouraged to reevaluate PEEP level during PP to avoid overdistension. Further studies, including a randomized–controlled trial, are now warranted to confirm potential PP benefits during ECMO.

## Supplementary information

**Additional file 1.** Details about exclusion criteria, EIT data acquisition and analysis, and exclusion of the out-of-phase regions.

## Data Availability

The datasets used and analyzed during the current study are available from the corresponding author on reasonable request.

## Abbreviations

ARDS: Acute respiratory distress syndrome
ECMO: Extracorporeal membrane oxygenation
EELI: End-expiratory lung impedance
EIT: Electrical impedance tomography
ICU: Intensive care unit
PP: Prone positioning
PEEP: Positive end-expiratory pressure
ROI: Regions of interest
SP: Supine positioning
VT: Tidal volume
VV: Veno-venous
VILI: Ventilator-induced lung injury
