# Supplementary material for: Prone positioning monitored by electrical impedance tomography in patients with severe acute respiratory distress syndrome on veno-venous ECMO
Source: Ann Intensive Care. 2020 Feb 3;10:12. doi: 10.1186/s13613-020-0633-5 (PMC6997307; doi:10.1186/s13613-020-0633-5)
Supplement: Supplementary file 1 — Additional file 1. Details about exclusion criteria, EIT data acquisition and analysis, and exclusion of the out-of-phase regions. [file 13613_2020_633_MOESM1_ESM.docx]

**Supplemental Digital Content**

**Prone positioning monitored by electrical impedance tomography in patients with severe acute respiratory distress syndrome on veno-venous ECMO**

**Guillaume Franchineau, MD ^1,2^, Nicolas Bréchot, MD, PhD ^1,2^, Guillaume Hekimian, MD ^1,2^, Guillaume Lebreton, MD, PhD ^1,3^, Simon Bourcier, MD ^1,2^, Pierre Demondion, MD ^1,3^, Loïc Le Guennec, MD ^1,2^, Ania Nieszkowska, MD ^1,2^, Charles-Edouard Luyt, MD, PhD ^1,2^, Alain Combes, MD, PhD ^1,2^, and Matthieu Schmidt, MD, PhD ^1,2^**

**Exclusion criteria**

- Contraindications of Electric Impedance Tomography (EIT): pacemaker, automatic implantable cardioverter defibrillator, contraindications to thoracic belt placement (e.g., thoracic or spinal cord trauma, recent thoracic surgery)
- Contraindications of high Positive End-Expiratory Pressure (PEEP) levels: undrained pneumothorax, bronchopleural fistula, or hemodynamic instability (i.e., use of intravenous fluids or vasopressors >2 mg/h of norepinephrine or 0.5 mg/h of epinephrine)
- Contraindications of Prone Positioning (PP): facial, vertebral, or pelvic fracture, intracranial hypertension, haemoptysis, tracheal surgery
- Or pregnancy

**EIT data acquisition and analysis**

During the protocol, a silicone EIT belt, with 16 surface electrodes, was placed around the patient’s thorax in one transversal plane corresponding to the sixth intercostal parasternal space, then connected to the EIT monitor (Pulmovista, Dräger) for bedside visualization. EIT data were generated by application of a small alternating electrical current (5 mA at 50 kHz). For each patient we used the same baseline reference. EIT data were continuously visualized on the Pulmovista screen during the whole protocol without modification of the belt. Only the last 5 minutes of each step were recorded and saved in a single EIT patient file.

EIT images were continuously recorded at 20 Hz during the last 5 minutes of each step. The data were digitally filtered using a low-pass filter with a cut off frequency of 40/min to eliminate small impedance changes synchronous with the heart rate. Lung images were divided into two symmetrical non-overlapping ventral-to-dorsal horizontal regions of interest (ROIs) ^1^: ventral region and dorsal region. The vertical height of these ROIs was identical and corresponded to 50% of the anteroposterior diameter. The EIT scans consist of images showing impedance with a 32X32 color-coded matrix. Output pixel values represented changes in local impedance, between the end of expiratory and inspiratory. Δz at each step was defined as the difference between end-inspiratory lung impedance and end-expiratory lung impedance. These variations were visualized on functional EIT images showing Δz per pixel averaged over 1 minute. Δz is known as well-correlated with tidal volume ^1^. In addition, because the driving pressure was constant herein, Δz changes was also correlated to local compliance variation ^2^. Distribution of EIT tidal ventilation in a ventral to dorsal direction was expressed according to the VT_dorsal_/VT_global_ ratio ^3–5^. The VT_dorsal_/VT_global_ ratio was calculated by dividing the dorsal tidal impedance variation by the total tidal impedance variation. Local changes of impedance during PP allowed estimation of regional change of compliance. At each step of PP session, local change of compliance was compared to baseline (before PP), and a compliance win and compliance loss was expressed as a percentage deviation. For instance, a local compliance win of 50% and a compliance loss of 25% compared to baseline illustrate a 25% compliance win at the thoracic belt level.

At each step, we recorded the End-Expiratory Lung Impedance (EELI), for the entire lung and at each ROI, which were well correlated to global and regional lung volumes, respectively ^6^. Thus, EELI change reflected end-expiratory lung volume with its distribution visualized on the EIT screen. Lastly, EIT data were stored and analysed offline on a personal computer. In order to estimate “optimal PEEP” in supine and PP, the PEEP trials were recorded and analysed offline, with an estimation of the overdistention and the collapse % as previously described ^2,7^. “Optimal PEEP” was identified as the PEEP level allowing both the lowest value of overdistention and collapse. The optimal PEEP estimated on supine position was applied during all the protocol. Another identical PEEP trial was performed at the end of the PP session, to compare “optimal PEEP” obtained in supine and in prone.

**Exclusion of the out-of-phase regions:**

Out-of phase regions were due to pleural effusions and/or heart region. These regions were deleted from the analysis. The % of these zones over the total impedance are reported in the Table below.

|  | Before PP | PP H_0_ | PP H_1_ | PP H_6_ | PP H_14_ | PP H_16_ | SP H_0_ | SP H_6_ |
| --- | --- | --- | --- | --- | --- | --- | --- | --- |
| Number of PCG+ patients | 2 | 7 | 6 | 7 | 6 | 6 | 3 | 2 |
| Percentage of the total impedance | 15 | 9 | 8 | 5 | 6 | 5 | 6 | 9 |
| Number of PCG- patients | 5 | 5 | 5 | 5 | 6 | 6 | 7 | 6 |
| Percentage of the total impedance | 26 | 17 | 15 | 14 | 19 | 9 | 16 | 12 |

**References**

1. Victorino JA, Borges JB, Okamoto VN, Matos GFJ, Tucci MR, Caramez MPR, Tanaka H, Sipmann FS, Santos DCB, Barbas CSV, Carvalho CRR, Amato MBP: Imbalances in regional lung ventilation: a validation study on electrical impedance tomography. Am J Respir Crit Care Med 2004; 169:791–800

2. Costa ELV, Borges JB, Melo A, Suarez-Sipmann F, Jr CT, Bohm SH, Amato MBP: Bedside estimation of recruitable alveolar collapse and hyperdistension by electrical impedance tomography. Intensive Care Med 2009; 35:1132–7

3. Bikker IG, Preis C, Egal M, Bakker J, Gommers D: Electrical impedance tomography measured at two thoracic levels can visualize the ventilation distribution changes at the bedside during a decremental positive end-expiratory lung pressure trial. Crit Care 2011; 15:R193

4. Frerichs I, Dargaville PA, Genderingen H van, Morel DR, Rimensberger PC: Lung Volume Recruitment after Surfactant Administration Modifies Spatial Distribution of Ventilation. Am J Respir Crit Care Med 2006; 174:772–9

5. Luepschen H, Meier T, Grossherr M, Leibecke T, Karsten J, Leonhardt S: Protective ventilation using electrical impedance tomography. Physiol Meas 2007; 28:S247–60

6. Bikker IG, Leonhardt S, Bakker J, Gommers D: Lung volume calculated from electrical impedance tomography in ICU patients at different PEEP levels. Intensive Care Med 2009; 35:1362–7

7. Franchineau G, Bréchot N, Lebreton G, Hekimian G, Nieszkowska A, Trouillet J-L, Leprince P, Chastre J, Luyt C-E, Combes A, Schmidt M: Bedside Contribution of Electrical Impedance Tomography to Setting Positive End-Expiratory Pressure for Extracorporeal Membrane Oxygenation-treated Patients with Severe Acute Respiratory Distress Syndrome. Am J Respir Crit Care Med 2017; 196:447–57
